# Innovative Approaches in Microtia Treatment: Advancements in Tissue Engineering and Scaffold Design

**DOI:** 10.1007/s10439-025-03851-7

**Published:** 2025-09-23

**Authors:** Jael Adrián Vergara-Lope Núñez, Juan Moisés Ocampo-Godínez, Febe Carolina Vàzquez-Vàzquez, Armando Apellaniz-Campo, Edgar Oliver Lopez Villegas, Marco Antonio Álvarez-Pérez

**Affiliations:** 1 Laboratorio de Bioingeniería de Tejidos, División de Estudios de Posgrado e Investigación (DEPeI), Facultad de Odontología, Universidad Nacional Autónoma de México, Ciudad Universitaria, Circuito Exterior s/n, Coyoacán, 04510 Mexico City, Mexico; 2 Central de Microscopia, Escuela Nacional de Ciencias Biológicas, Instituto Politécnico Nacional, Mexico City, Mexico; 3 Infection Immunity and Inflammation Research and Teaching Department, University College London Institute of Child Health, London, UK; 4 Laboratorio de Materiales Dentales, DEPeI, School of Dentistry, Universidad Nacional Autónoma de México, Ciudad Universitaria, Circuito Exterior s/n, 04510 Ciudad de México (CDMX), Mexico; 5 División de Cirugía Plástica y Reconstructiva, Hospital General “Dr. Manuel Gea Gonzalez”, Mexico City, Mexico

**Keywords:** Microtia, Translational medicine, Tissue engineering, Nagata surgery, Regenerative medicine, Biodevices

## Abstract

Facial symmetry is paramount in societal perceptions of attractiveness, with symmetric faces receiving higher ratings. This is particularly relevant for individuals with microtia, a congenital condition affecting external ear formation, who often experience psychosocial challenges such as anxiety and depression. Auricular prostheses and High-density porous polyethylene (MEDPOR^®^) offer an aesthetic solution. However, they are related to disadvantages like color mismatches, periodic replacement, and skin infections. Currently, the Nagata technique, regarded as the "gold standard" for microtia treatment, involves a two-step surgical procedure using autologous rib cartilage to reconstruct the auricle. Despite its widespread use, this method is highly invasive and associated with significant risks, including chronic pain, skin necrosis, and variable aesthetic outcomes dependent on the surgeon’s skill. Tissue engineering presents a novel approach to microtia treatment, focusing on three core principles: creating a temporary scaffold for cellular support, selecting appropriate cells for seeding, and optimizing the regeneration process through molecular enhancements. This review discusses a novel perspective for microtia treatment with innovative methodologies that seek to improve aesthetic and functional outcomes, mainly through advancements in tissue engineering and scaffold fabrication techniques.

## Introduction

Microtia is recognized as the most prevalent congenital malformation of the ear. According to the International Microtia and Atresia Workgroup, microtia is defined as a small and malformed auricle, often associated with stenosis or atresia of the external [1]. Microtia ranks among the top ten congenital disabilities, with a prevalence of 1 case per 5500 to 26,000 live births [2, 3]. Auricular malformations may occur as isolated conditions or as part of a broader spectrum of syndromes, ranging from mild abnormalities to total absence [4].

The etiology of microtia remains largely unknown; however, certain perinatal risk factors have been identified as predictors of the condition [5]. These factors include multiple births, maternal age of 35 years or older, residence in urban areas, male sex, birth weight of less than 2500 g, gestational age of less than 38 weeks, and a higher prevalence among Hispanic and Asian populations [6–10]. Additionally, genetic factors have been well-documented in association with various syndromes, such as mandibular dysostosis, Meier–Gorlin syndrome, Goldenhar syndrome, Treacher-Collins syndrome, and chromosomal anomalies like trisomy 18, 21, and 22 [3–5, 11, 12].

**Fig 1.**
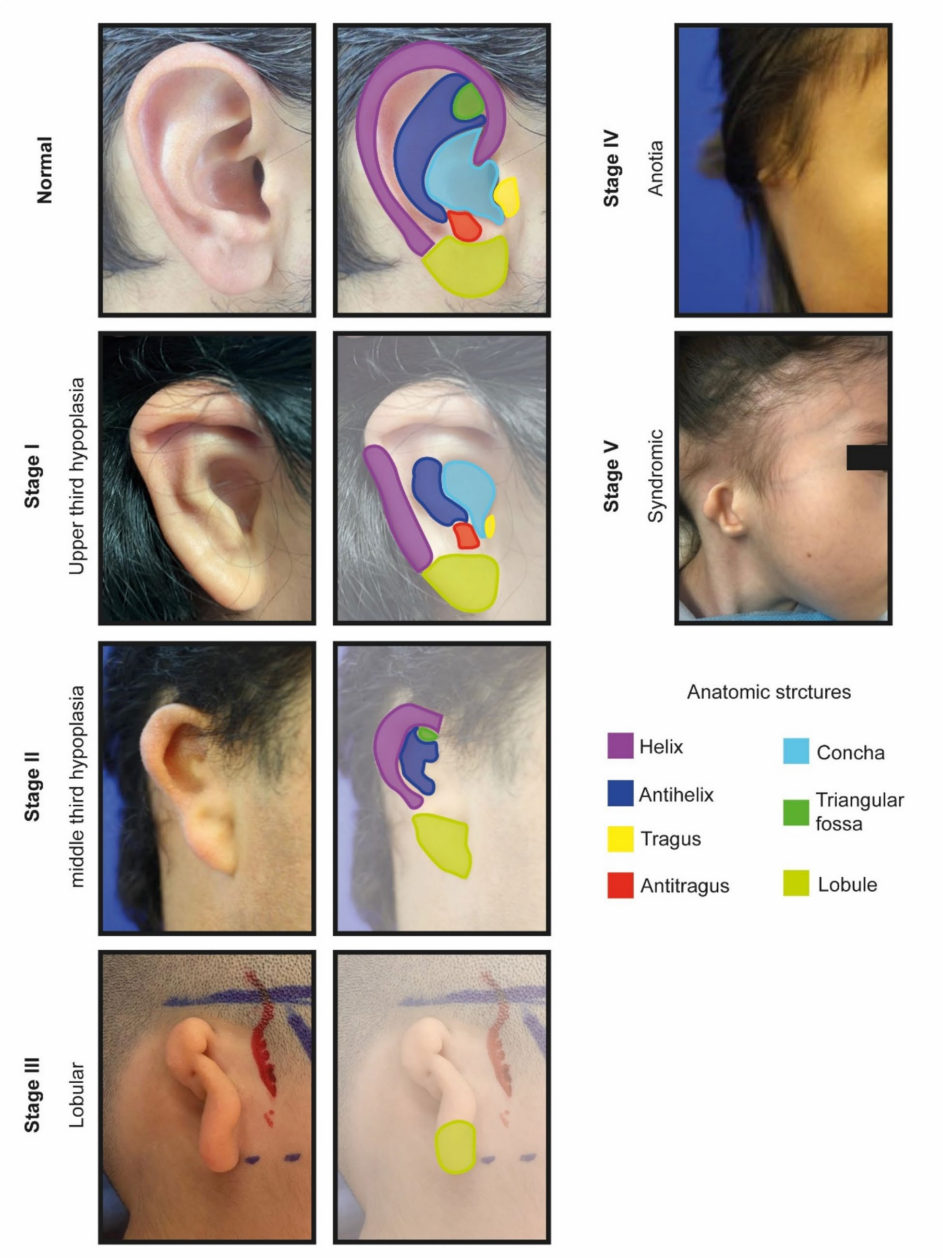
The Apellaniz classification system. To improve surgical correction outcomes, this classification includes Type I (upper third hypoplasia), Type II (middle third hypoplasia), Type III (lobular or complete hypoplasia), Type IV (anotia or complete aplasia), and Type V (dystopia).

Currently, there is no universal classification system for microtia. However, several classifications provide different perspectives, helping to stratify microtia better and improve treatment options (Table 1). The Marx classification categorizes microtia into four grades: Grade I (slightly smaller ear), Grade II (smaller with underdeveloped or absent structures), Grade III (cartilage vestige), and Grade IV (anotia) [13, 14]. In contrast, Tanzer’s classification offers a more detailed approach with five types, ranging from anotia (Type I) to prominent ears (Type V), incorporating aspects like ear canal permeability. Nagata’s classification focuses on surgical needs, categorizing patients based on the specific anatomical structures requiring reconstruction. However, it lacks a clear stratification of severity, which can lead to confusion. The Apellaniz classification system combines the surgical requirements and clinical presentations of microtia to categorize the condition (Fig. 1). This classification comprises five categories and correlates the required cartilage structure for reconstruction. Notably, it includes syndromic microtia, a variant that necessitates a distinct surgical approach for the lower auricular implantation observed in these patients. [15]. Lastly, the HEAR MAPS system presents a multidisciplinary approach, integrating anatomical, functional, and radiological alterations, reflecting the complexity of microtia [16].

**Table 1.** Comparison of Microtia Classifications. Comparison between different classification systems for microtia

Comparison of Microtia Classifications				
Marx	Tanzer	Nagata	Apellaniz	HEAR MAPS
**Grade I:** Slightly smaller auricle, but all structures are present. **Grade II:** Smaller auricle with underdeveloped or absent structures. **Grade III:** Only a remnant cartilage is present. **Grade IV:** Snotia.	**Type I:** Anotia. **Type II:** Complete hypoplasia. a: with external auditory canal atresia. b: without external auditory canal atresia. **Type III:** Middle third hypoplasia. **Type IV:** Upper third hypoplasia. a: constricted ear (cup or lop) b: criptotia. c: upper third hypoplasia. **Type V:** Prominentears.	**Lobular:** Malpositioned cartilage remnants and lobules. Lacks conchae, meatus, or tragus. **Conchal:** Conchae, tragus, and antitragus present. **Small conchae:** Malpositioned remnants and lobule, a small indentation in the conchae. **Anotia:** Total absence or small cartilage remnant. **Atypical:** Not classifiable in another category.	**Stage I:** Upper third hypoplasia. The reconstructive requirements are the helix, crurae, and antihelix **Stage II:** Middle third hypoplasia. The reconstructive requirements are the helix, crurae, antihelix, and antigragus. **Stage III:** Lobular The reconstructive requirements include helix, crurae, antihelix, antigragus, incisura, and tragus. Stage IV: Anotia. All the structures need to be reconstructed. **Stage V:** Dystopia(syndromic). All the structures need to be reconstructed.	**Hear bone/air (PTA2 dB HL):** Bone PTA2/Air PTA2. **Ear (microtia):** Grade 1 (Normal). Grade 2 (Mild malformation). Grade 3 (Moderate malformation). Grade 4 (Anotia). **Atresia Jahrsdoerfer CT scale:** Grade 1–10. **Remnant earlobe:** Grade 1 (Normal). Grade 2 (Mildly reduced). Grade 3 (Moderately reduced). Grade 4 (Severely reduced/absent). **Mandible:** Grade 1 (Normal). Grade 2 (Mildly reduced). Grade 3 (Moderately reduced). Grade 4 (Severely reduced/absent). **Asymmetry of soft tissue:** Grade 1 (Normal). Grade 2 (Mildly reduced). Grade 3 (Moderately reduced). Grade 4 (Severely reduced/absent). **Paresis of the facial nerve House-Brackmann scale:** Grade 1–6 **Syndrome:** Grade 1 (None) Grade 2 (Yes)

## Current Perspectives on Microtia Treatment

**Fig 2.**
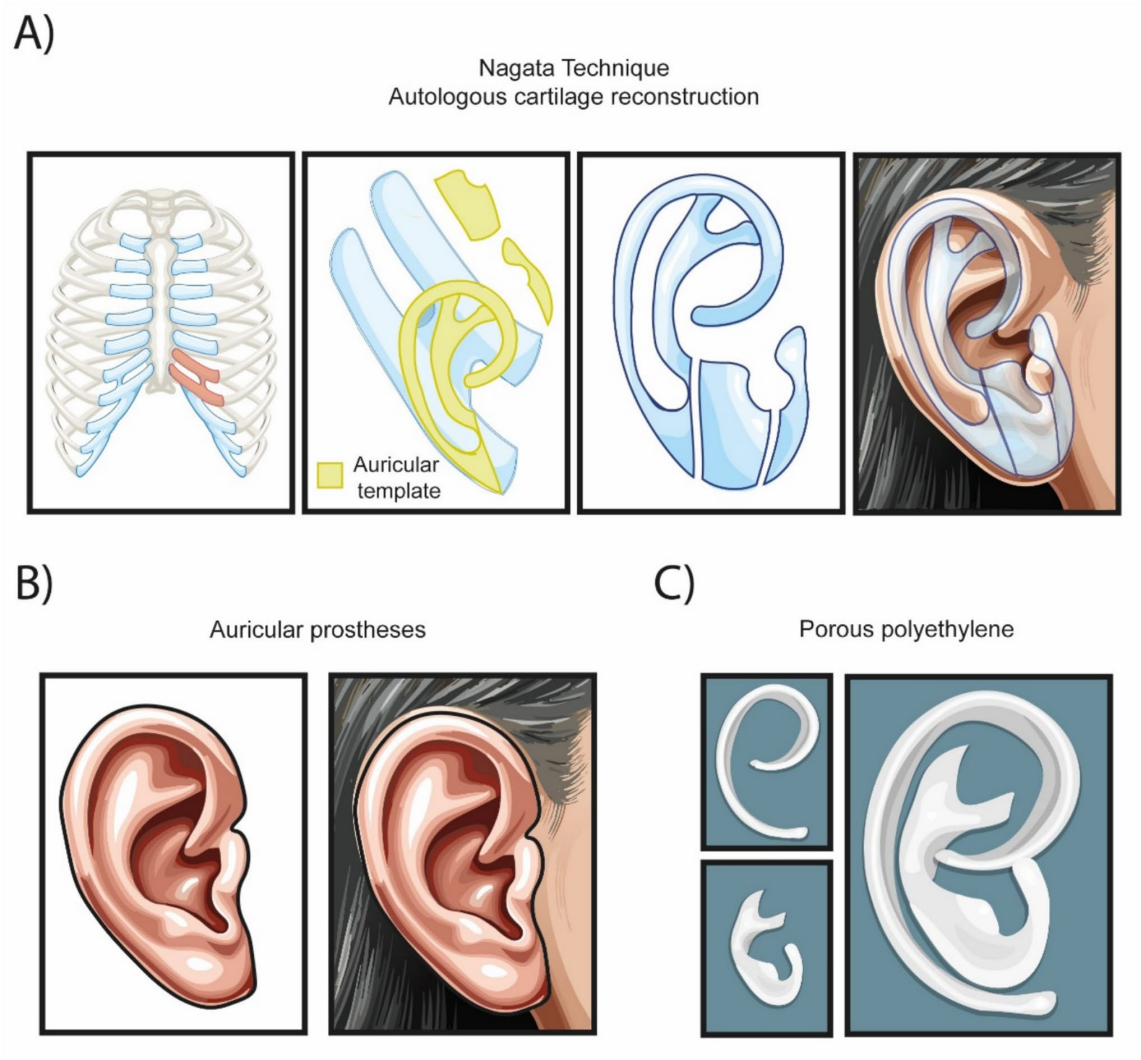
Current Microtia Treatments. **A** In the Nagata technique, costal cartilage is harvested and shaped using a template derived from the contralateral ear to resemble a natural ear, which is subsequently implanted. **B** Conventional auricular prostheses replicate the morphology of the ear but often exhibit a color mismatch compared to the surrounding tissue. **C** Porous polyethylene frameworks.

Facial symmetry is essential in contemporary society, with symmetric faces receiving the highest attractiveness ratings. Minimal deviations in proportions are often perceived as less attractive [17]. Consequently, patients with microtia frequently confront psychosocial challenges, including anxiety, depression, and feelings of loneliness [18]. The Nagata technique is widely regarded as the "gold standard" for treatment (Fig. 2A). This two-step procedure utilizes autologous cartilage harvested from the ribs to reconstruct the ear [19].

The first step involves obtaining and shaping an autologous rib graft to replicate the primary structures of auricular anatomy. Our group employs a 6th over 7th surgical technique for this purpose. Briefly, a contralateral surgical approach is utilized to harvest the 6th, 7th, and 8th costal cartilages while preserving the visceral portion of the synchondrosis between the 6th and 7th cartilages. The natural curvature of the 7th cartilage facilitates bending over the 6th cartilage, which reduces surgical time and preserves the 9th costal cartilage [20]. The second step entails separating and elevating the auricular implant from the head to achieve a more realistic shape [21].

However, Nagata and other autologous cartilage reconstruction techniques are not without their drawbacks. It is considered a highly invasive procedure associated with a significant risk of chronic pain at the surgical site, flat skin necrosis, framework exposure, rib architecture collapse, infection, and the persistent risk of reabsorption [22]. Furthermore, the aesthetic outcomes of this technique can be variable and heavily dependent on the surgeon’s skill, resulting in excellent results in some cases while yielding poor outcomes in others [23]. As a result, new methodologies for treating microtia have emerged in recent years

## Auricle Prosthetic Through Implantology

Auricular prostheses represent another viable treatment option for microtia (Fig. 2B). These prosthetics provide an aesthetic solution for ear abnormalities resulting from trauma, cancer, or congenital conditions [24]. Advances in technology, including 3D scanning, computer-aided design (CAD), and computer-aided manufacturing (CAM), have significantly improved the accuracy of auricular prostheses [25]. An essential step in the use of prostheses is the removal of microtia cartilage. This involves meticulously excising all residual cartilage from the patient to ensure the area is completely smooth. Anchors are then affixed to the temporal bone, extending through the skin to secure the prosthesis in place. A mirror model of the contralateral ear is subsequently created and attached to these anchors using magnetic components, facilitating a stable connection between the skull and the external device. [26, 27]. While using these prostheses generally yields high patient satisfaction rates, several significant considerations must be considered. For instance, the area surrounding the anchor often exhibits serous fluid drainage, leading to unpleasant odors. Therefore, rigorous cleaning and maintenance of the prosthesis are essential for patient comfort. Moreover, to ensure the longevity of the prosthesis, patients are advised to remove it during sports activities and at night, and patients must exercise caution with jaw movements to prevent dislodgement. Additionally, prostheses typically require replacement every 2–3 years due to color mismatch, underscoring the ongoing maintenance involved in their use [27, 28]. While these prosthetic solutions provide significant benefits, they also necessitate a commitment to proper care and regular follow-ups.

## Alternative Treatments Looking for a Better Aesthetic Result

In the past century, high-density porous polyethylene (Fig. 2C) has emerged as a promising therapeutic alternative for auricular reconstruction in cases of microtia, marketed under the MEDPOR^®^ brand [29]. A significant advantage of this synthetic, biocompatible material is eliminating the need for autologous cartilage harvesting, thereby reducing the risks associated with thoracic surgery. Furthermore, bone bridge implantation can be performed concurrently with auricular reconstruction, addressing any hearing impairments associated with microtia [30]. Despite these advantages, the application of MEDPOR^®^ implants is not without complications. The implantation technique necessitates multiple surgical interventions to cover the device with a flap and skin graft, which can lead to local skin loss, infection, and device extrusion [31]. Sometimes, skin grafts may be harvested from the abdomen or neck to cover the implant surface [32]. These factors contribute to the trauma of the reconstruction process, and the long-term outcomes of such procedures remain uncertain [27–30].

To enhance the performance of these devices, a tissue engineering approach has been employed to coat MEDPOR^®^. Preliminary studies in animal models indicate that the combination of elastic cartilage and MEDPOR^®^ yields promising results, potentially reducing side effects and improving outcomes in future clinical applications [29]. This innovative strategy may pave the way for more effective and less invasive treatments for microtia, ultimately improving the quality of life for affected individuals.

## Tissue Engineering as an Innovative Solution for Microtia

The principles of tissue engineering are fundamentally straightforward. Most protocols for tissue regeneration within this biomedical field adhere to three primary characteristics. Firstly, a temporary structure is required to support cell attachment, proliferation, and metabolic activity [33, 34]. Material selection must consider the mechanical properties, physicochemical composition, and microarchitectural structure of the natural tissue intended for regeneration [35, 36]. Secondly, the choice of appropriate cells for seeding is crucial. A thorough understanding of tissue histology and, most importantly, the functional requirements for regeneration are vital for effectively selecting and seeding cells within scaffolds. The primary objective is to position the correct cells within the appropriate scaffold, enabling the cells to attach to the material, proliferate, produce extracellular matrix (ECM) proteins, and ultimately facilitate regeneration [37, 38]. Thirdly, molecular enhancement may be employed to optimize the process. Scaffolds can enhance cellular function or promote cell differentiation by incorporating nutrient concentrations, differentiation molecules, growth factors, signaling factors, and other components [39, 40].

In 1997, Cao et al. successfully engineered cartilage in the shape of a human ear. The researchers designed a scaffold resembling a human ear, which was seeded with chondrocytes derived from calf articular cartilage and subsequently transplanted onto the backs of nude mice. The chondrocytes demonstrated the ability to grow and synthesize ECM, marking a revolutionary discovery in the field [41]. Since then, tissue engineering has emerged as a promising avenue for regenerating elastic cartilage [42–44].

Numerous in vitro and vivo studies concerning auricle engineering have evidenced the regeneration of auricular shape and cartilage histology; however, none have successfully maintained auricular structure over time [42, 45]. Consequently, it is imperative to explore the development of novel tissue engineering techniques that facilitate the long-term preservation of auricular morphology. We will discuss several pertinent aspects that influence auricular regeneration in this context.

## Anatomical Challenges: Navigating the Complexity of Ear Architecture

**Fig 3.**
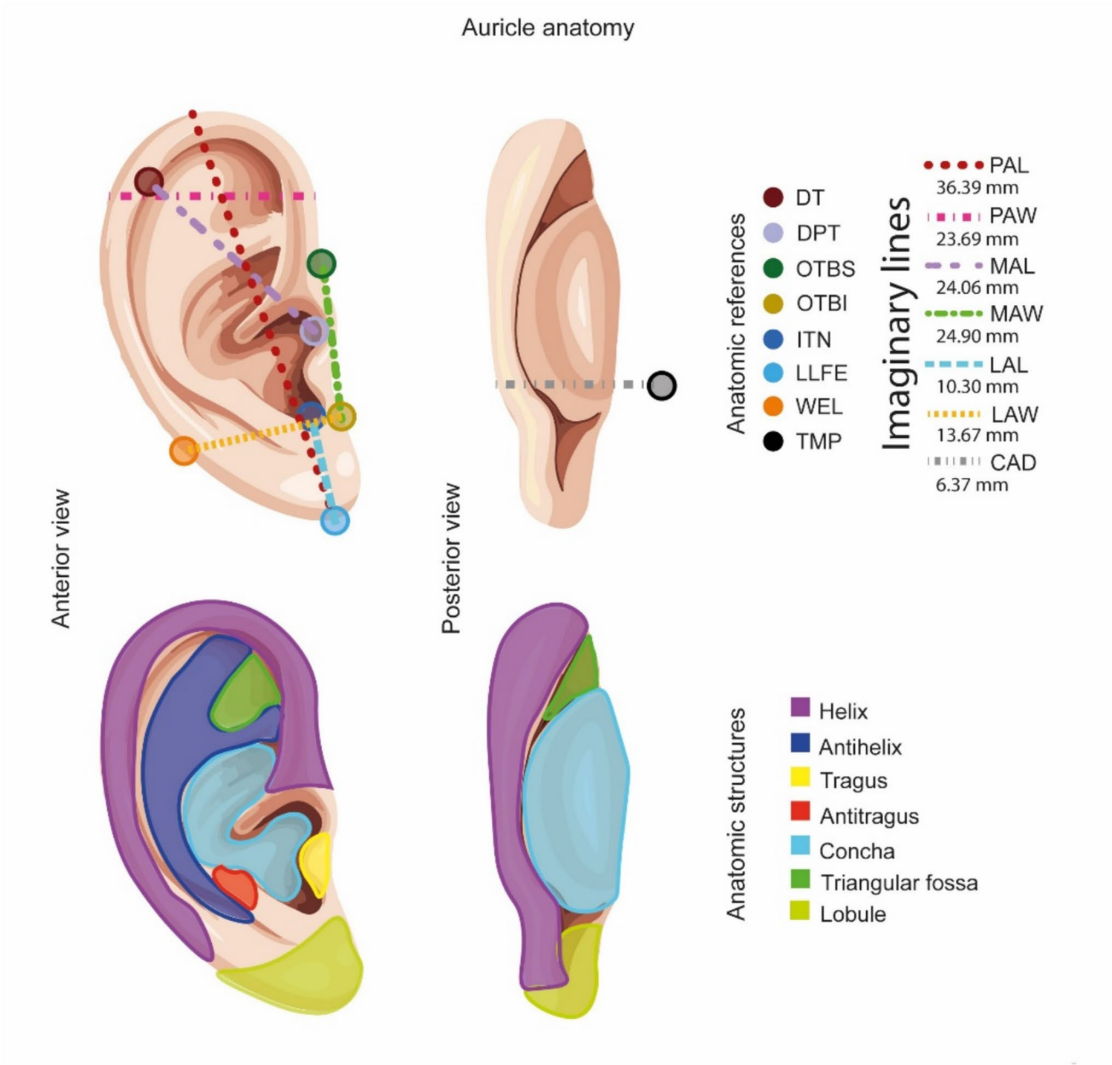
Normal Auricle Anatomy. Schematic representation of the most significant prominences, depressions, reference lines, and points of the normal auricular anatomy in an anterior (left) and posterior view (right). Physiognomic Auricular Length; PAL. Physiognomic Auricular Width; PAW. Morphological Auricular Length; MAL. Morphological Auricular Width; MAW. Lobule of Auricular Length; LAL. Lobule of Auricular Width; LAW. Cephaloauricular Distance; CA. Darwin’s tubercle; DT. Deepest point in tragus; DPT. Otobasion superior; OTBS. Otobasion inferior; OTBI. Intertragic notch; ITN. Lowest lobule free edge; LLFE. Widest edge of the lobule; WEL. Temporal mastoid process; TMP

As previously mentioned, the anatomy of the auricle is notably intricate (Fig. 3). Traditionally, the auricle is described as a thin, oval, and elastic structure characterized by various prominences and depressions. The auricular surface comprises the helix, antihelix, tragus, and antitragus, alongside three depressions known as the scaphoid fossa, triangular fossa, and concha [4, 15]. For a more precise description, seven imaginary lines are drawn and utilized as references to delineate the ear and its relationship with the head.

In neonates, the longest line extends from the top of the ear to the bottom, with a mean distance of 36.39 mm, referred to as the Physiognomic Auricular Length (PAL). Orthogonal to this line, the Physiognomic Auricular Width (PAW), which measures a maximum of 23.69 mm, spans from the preauricle to the postauricle [4, 46]. The distance between the tragus and Darwin’s tubercle (DT) and the deepest point in the tragus (DPT) is termed the Morphological Auricular Length (MAL), averaging 24.06 mm [4, 47]. The Morphological Auricular Width (MAW) is measured between otobasion superior (OTBS) and inferior (OTBI), with this 24.9 mm line extending from the superior ear attachment point to the lower attachment of the lobule [4, 48]. The lobule serves as a reference for two additional lines: the Lobule of Auricular Length (LAL), which runs from the intertragic notch (ITN) to the lowest lobule free edge (LLFE), and the Lobule of Auricular Width (LAW), which extends from the OTBI to the most comprehensive edge of the lobule (WEL). Both lines average 10.3 mm and 13.67 mm, respectively [4, 49]. The Cephaloauricular Distance (CAD), measuring 6.37 mm, is the linear distance between the helix and the temporal mastoid process (TMP). The Auriculocephalic Angle, representing auricular projection, typically exhibits a 4.64° inclination relative to the scalp [4, 49, 50]. The auricle undergoes rapid development, reaching 90% of its total length within the first year of life. By age 10, the average dimensions are approximately 59.0 × 32.5 mm in females and 63.5 × 35.3 mm in males, achieving 99% of the final dimensions [49, 51]. Consequently, an ideal scaffold should replicate the prominences, depressions, widths, lengths, and angles in the contralateral ear to enhance facial symmetry [42, 45, 50].

## Histological Insights: Understanding the ECM of Elastic Cartilage

**Fig. 4.**
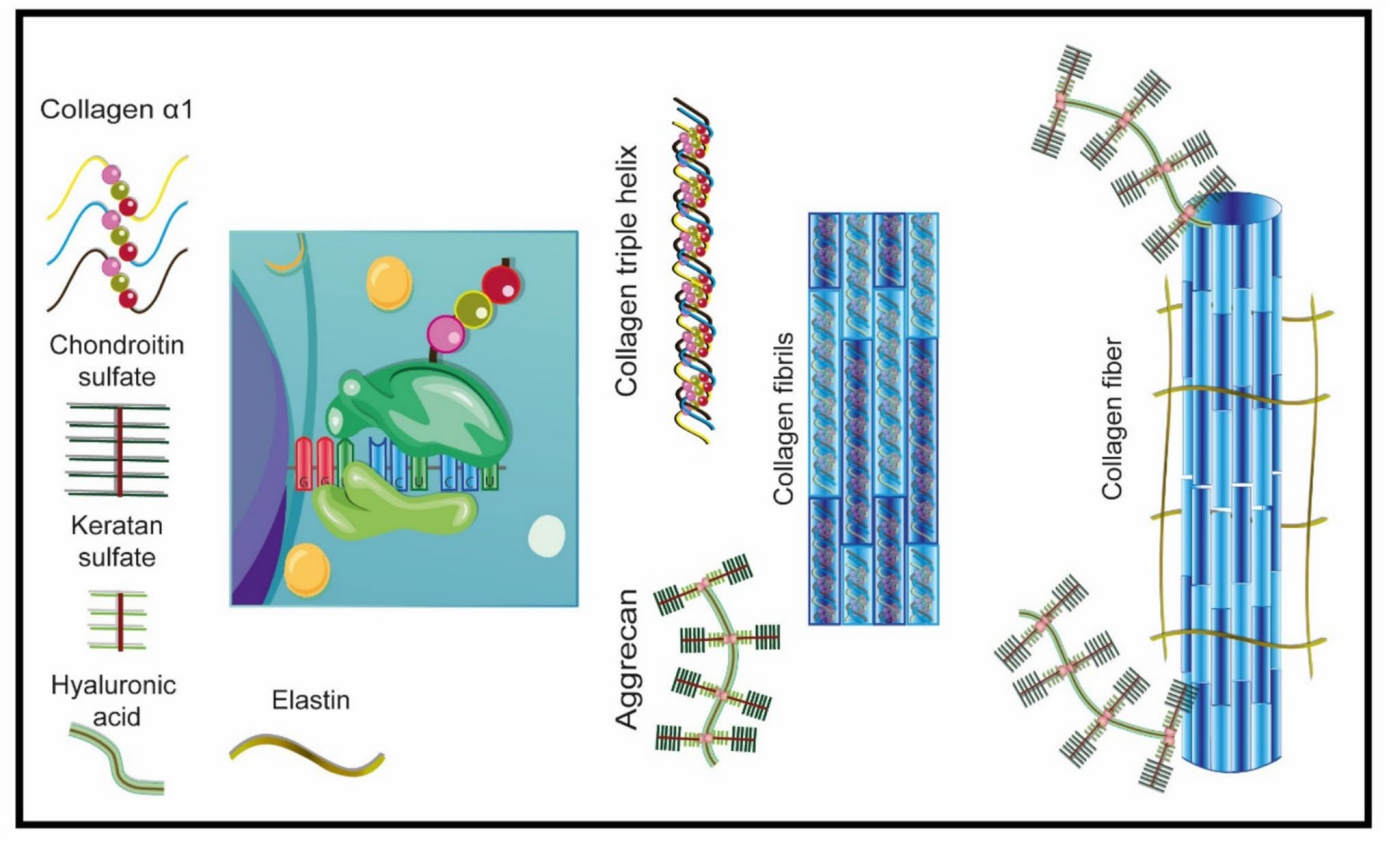
Extracellular matrix formation. The formation of the extracellular matrix of elastic cartilage begins with the synthesis of an alpha 1 chain of collagen which joins two more chains to form a triple helix. The union of triple alpha 1 chains forms type 2 collagen fibers. On the other hand, the union of chondroitin sulfate with keratan sulfate along a hyaluronic acid core forms aggrecans. The union of collagen fibers, aggrecan and elastin form the main proteins of elastic cartilage

Cartilage is a specialized form of connective tissue. Depending on the proteins that constitute the extracellular matrix (ECM), cartilage is categorized into three types: hyaline, fibrous, and elastic. [52, 53]. Auricular cartilage (Fig. 4) is classified as elastic cartilage and contains ECM proteins that serve specific functions essential for proper operation [54]. It comprises approximately 73% water, 15% collagen, 9% proteoglycans, 5% multi-adhesive proteins, and 3–5% cells [55, 56]. Various types of collagens are present in cartilage; however, only types II, VI, IX, and X are considered specific to cartilage. Notably, collagen type II (Fig. 4), type I, and elastin constitute most of the fibers found in the ECM of elastic cartilage [52, 53]. Glycosaminoglycans, such as chondroitin sulfate and keratan sulfate, are significant components that combine to form aggrecan.

This complex consists of 100 chondroitin sulfate chains and up to sixty molecules of keratan sulfate, resulting in an aggrecan with a molecular size of 250 kDa [57, 58]. This intricate anatomy generates distinct mechanical profiles. The average Young’s modulus of the auricle is 1.66 MPa, although this varies depending on the anatomical region. The highest modulus is observed in the concha (2.08 MPa) and antitragus (1.79 MPa), while the lowest modulus subestirpe is found in the antihelix (1.71 MPa), tragus (1.67 MPa), and helix (1.41 MPa) [59].

**Fig. 5.**
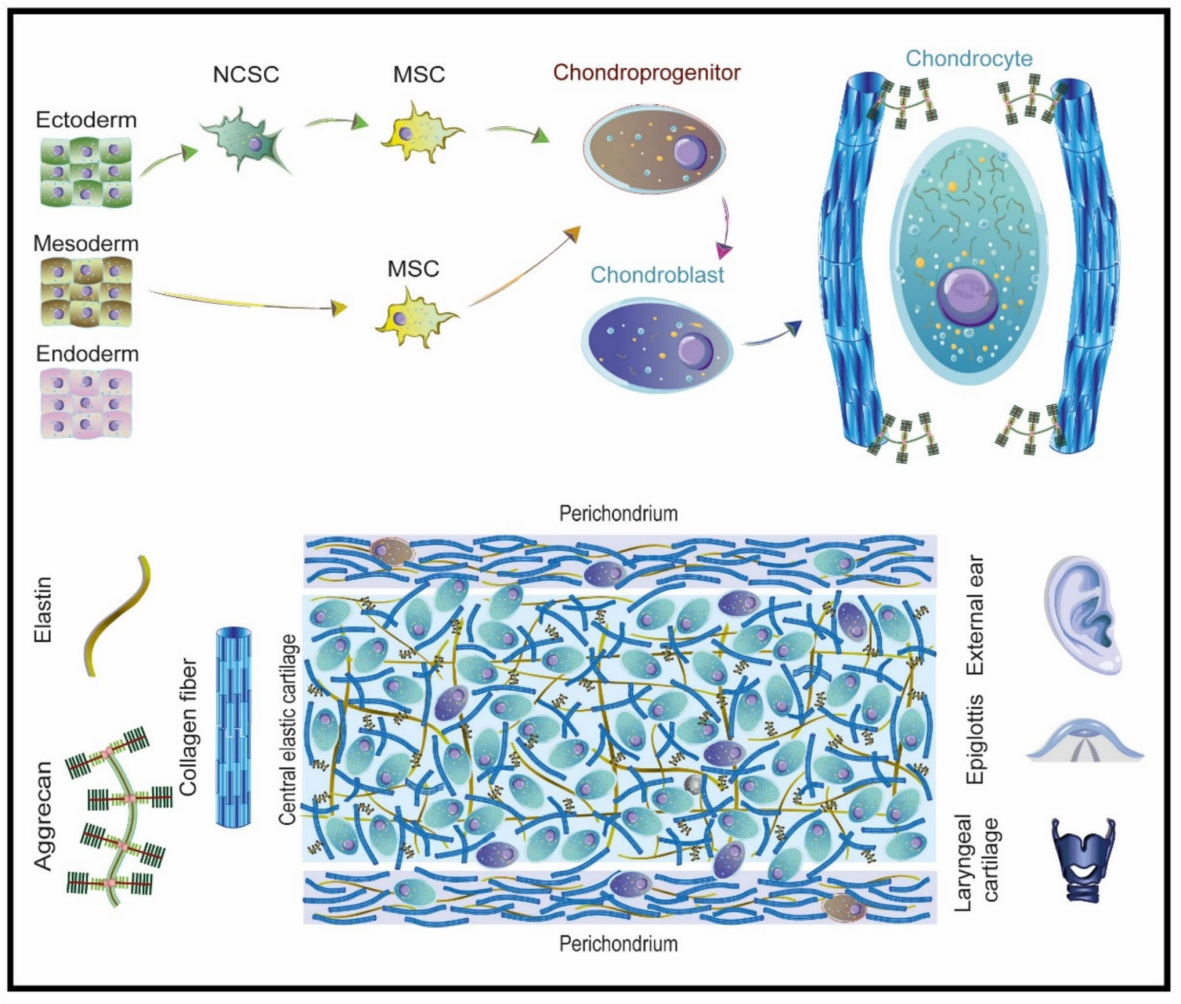
Elastic cartilage microenvironment. Upper: the process of differentiation from an embryonic state to a mature chondrocyte is illustrated. Lower: collagen fibers are interwoven with aggrecan and elastin fibers, forming lacunae in a mesh-like configuration. The perichondrium is depicted, containing chondroprogenitor cells and chondroblasts, while the central region displays mature chondrocytes and chondroblasts. Notably, the external ear, epiglottis, and laryngeal cartilage are specific regions where elastic cartilage is located

Traditionally, cartilage is classified as a mesoderm-derived tissue (Fig. 5), suggesting that chondrogenesis begins with mesenchymal stem cells (MSCs). These MSCs differentiate into chondroprogenitor cells, which exhibit hallmark characteristics of MSCs, including plastic adherence and the capacity for adipogenic, osteogenic, and chondrogenic differentiation. Chondroprogenitor cells express specific surface markers, such as CD44 + and CD90 + , indicating a high proliferative capacity [58, 60]. However, recent findings from single-cell RNA sequencing have revealed an ectodermal origin for auricular elastic cartilage. Specifically, neural crest cells have been shown to possess the potential to differentiate into mesenchymal cells, which subsequently mature into chondrocytes. This discovery suggests that auricular cartilage may not solely arise from mesodermal MSCs but may also be influenced by contributions from neural crest-derived progenitors. [61, 62]. Regardless of their embryological origin, chondroblasts and chondrocytes represent the terminal or mature stages of cartilage development. Chondrocytes, the predominant cell type within mature cartilage, are situated within a dense mesh of proteins known as lacunae and contribute to the organization, besides maintaining the ECM. The aggregation of these lacunae forms a complex three-dimensional architecture essential for cartilage function. Cartilage is an avascular structure, which means it does not contain blood vessels, and nutrients and oxygen do not arrive via capillaries. Also, cartilage has an aneural nature, which means the tissue does not have nerves, and the cartilage itself is insensitive to pain. Consequently, its nutrition depends on diffusion processes, which vary according to the type of cartilage. In articular cartilage, the primary nutrient source is the synovial fluid of the joint capsule; in fibrocartilage, nutrients diffuse from adjacent connective tissue and nearby blood vessels [63–66]. In elastic cartilage, a thin connective tissue layer known as the perichondrium fulfills this function [69]. Moreover, the limited regenerative capacity of the different cartilage types hinders effective tissue repair after injury [67–70]. Notably, some adverse outcomes observed during autologous cartilage reconstruction may be attributed to the origin of the cells involved. The disparity in embryological origins between elastic and hyaline cartilage may elucidate the histological mismatch that drives reconstructed ear resorption and mechanical properties alterations.

## What is the Optimal Source of Chondrocytes for Regenerating Auricular Scaffolds?

Having established the tissue context, the next step is identifying the ideal cell type. The most apparent choice is chondrocytes, with the primary source being residual cartilage obtained during autologous reconstruction for microtia. This relatively straightforward source provides homologous and autologous cells that theoretically mitigate immunological concerns [35, 37, 43, 44]. Genotypic comparisons between normal and microtia chondrocytes reveal a high degree of similarity, further supporting their use in regenerative protocols [71, 72]. However, it is essential to note that chondrocytes, including those from microtia cartilage, tend to de-differentiate in two-dimensional culture. This de-differentiation process affects all types of chondrocytes and alters their phenotypic profile, leading to an overexpression of type III collagen, a typical marker of this process [69]. Nevertheless, microtia chondrocytes have demonstrated the potential for phenotypic redifferentiation when cultured in a three-dimensional chondrogenic scaffold system [37, 71, 73].

An intriguing consideration is whether alternative sources of chondrocytes could be utilized. Various cartilaginous structures present potential options for cell procurement [74]. Chondrocytes harvested from the trachea, joints, nasoseptum, and ribs have been seeded in scaffolds to assess their regenerative capacities based on ECM production, gene expression, and cell viability [67, 73–75]. The results suggest that these sources may be viable for use in auricular scaffolds. However, it is crucial to recognize that the histology of elastic cartilage differs from that of most described sources, and chondrocytes from these alternative tissues may be predisposed to produce hyaline or fibrous ECM instead [74]. If this occurs, it could reintroduce some initial challenges associated with microtia reconstruction, such as loss of definition, increased rigidity, or implant resorption (Table 2).

**Table 2.** Strategies for Cartilage Reconstruction. This table provides a comprehensive overview of scaffold design and application, detailing each approach’s materials, cellular sources, and associated advantages and disadvantages. PVA: Poly(Vinyl Alcohol); PGA: Polyglycolic Acid; PLGA: Poly(DL-lactic-co-glycolic acid); PLA: Polylactic Acid; PCL: Polycaprolactone.

Manufacture method	Material	Cell source	Advantages	Disadvantages	Reference(s)
Bio-ink	Decellularized / Sodium alginate	Rabbit adipose-derived stem cells	Promoting cell adhesion, proliferation, and differentiation	Lack of auricular shape	81
Bio-ink	Type A gelatin/ Methacrylate	human adipose-derived mesenchymal stem cells	Expression of chondroitin sulfate, SOX9, and type II collagen	Lack of auricular shape	37
Hydrogel	GelMA	Healthy porcine chondrocytes	Boost endogenous elastin fiber production.	Not yet suitable for human use	53
Hydrogel	Chitosan/ Gelatin/ PVA	Human microtia	Maintenance of chondrogenic phenotype	Low extracellular matrix formation	43
Decellularized	Decellularized/ Chitosan	Rat annulus fibrosus stem cells	Neocartilage formation	Not elastic cartilage formation	78
Fibrillar	PGA/PLA	Human microtia	Redifferentiation to functional chondrocytes and neocartilage formation	Lack of auricular shape.	72
Fibrillar	PGA	Healthy and microtia human cartilage	Neocartilage formation	Lack of auricular shape	71
3D printing	PLA	Human auricular chondrocytes and bone marrow‐derived human mesenchymal stem cells.	Type II collagen formation	Lack of auricular shape	68
3D printing	PCL	Human microtia	Neocartilage formation	Lack of definition in anatomical structures	44
3D printing	PLA/ Decellularized	None	Preservation of anatomical structures	Fibrovascular tissue ingrowth	80
3D printing/ Fibrillar	PCL/ PLA/ PGA fibers	Human microtia	Neocartilage formation with clinical application	Materials with slow degradation	42
Coating implant	Medpor^®^/ PLGA	Healthy rabbit chondrocytes	Neocartilage formation and higher mechanical strength	Still use a non-biodegradable core	29

Additionally, undifferentiated cells may represent a promising alternative. MSCs or induced pluripotent stem cells are viable options for auricular regeneration. These cells possess self-renewal properties and, importantly, the capacity to differentiate into osteoblasts, adipocytes, and chondroblasts. Current findings indicate their ability to expand and differentiate within scaffolds; however, the methodologies involved remain costly, posing challenges for clinical application [37, 61, 68, 76].

## Scaffolds: Essential Tools in Regenerative Medicine

**Fig 6.**
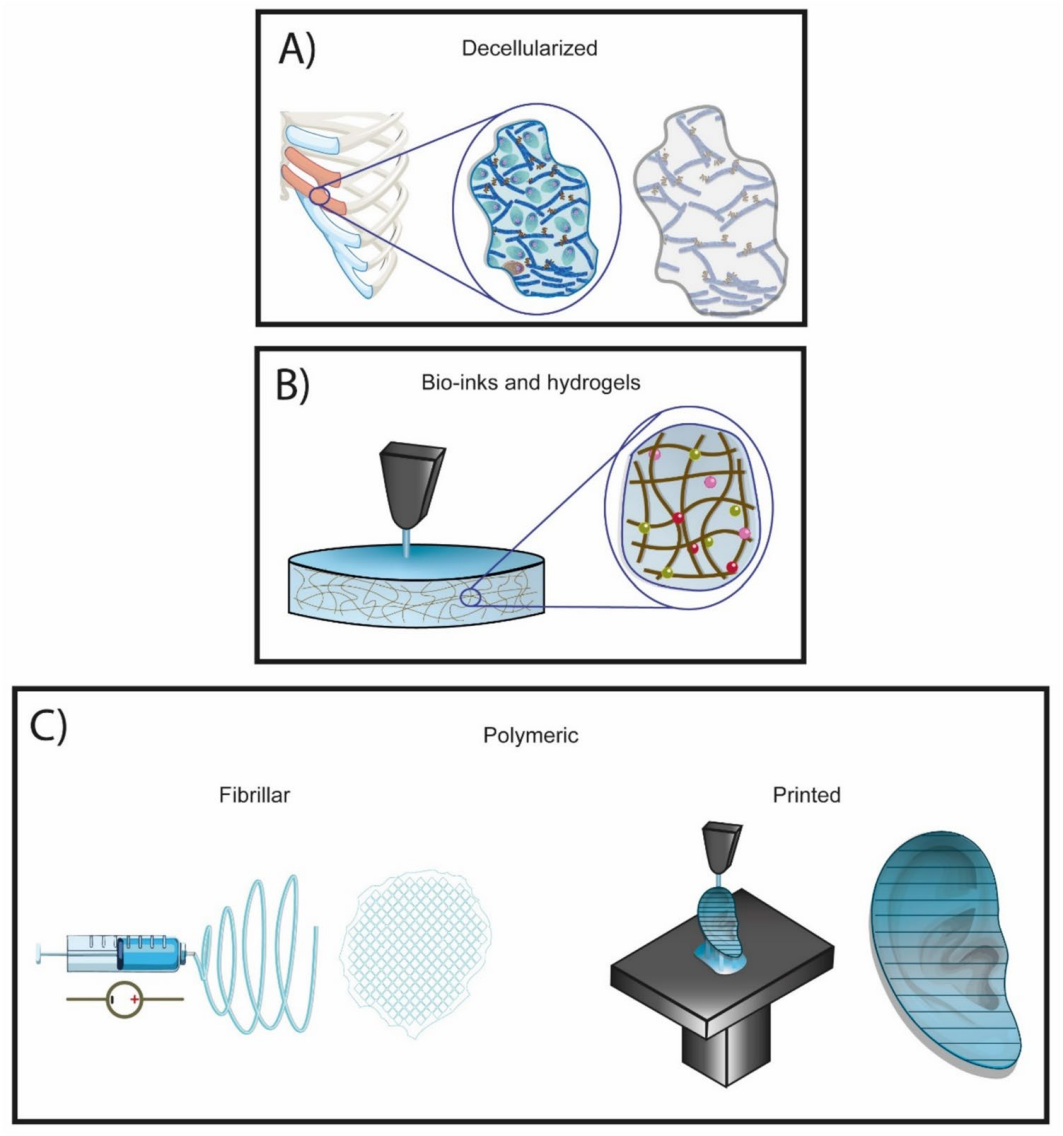
Tissue Engineering Scaffolds. Various techniques are employed to fabricate scaffolds for cartilage tissue regeneration. **A** Decellularized scaffolds involve the removal of resident cells while preserving the native extracellular matrix of the tissue. **B** Bio-inks and hydrogels are highly hydrated scaffolds that facilitate cell growth. **C** Polymeric scaffolds encompass a diverse array of manufacturing methods and materials, including air-jet spinning and fused deposition modeling (3D printing)

The selection of the scaffold is as critical as the choice of cells. Consequently, the scientific community explores various manufacturing techniques and a diverse range of materials to replicate the ECM of elastic cartilage effectively. For example, the decellularization (Fig. 6A) process involves the removal of all cellular components from the ECM. Through mechanical, chemical, or physical methods, native cells are eliminated while preserving specific local tissue properties. Retaining histological morphology, mechanical properties, ECM composition, and signaling molecules results in a scaffold primed for cell seeding from various sources, facilitating the regeneration of bioartificial organs [77, 78]. Furthermore, these characteristics enhance cellular performance due to native components within the decellularized scaffold, which may aid regeneration.

However, as previously noted, the unique anatomical variations of the auricle among individuals pose challenges; using ear tissue from a different donor may lead to mismatches in facial symmetry. A potential solution is to combine decellularized ECM with other manufacturing processes supported by CAD-CAM technologies [25, 79].

Following this, cell recellularization becomes the next critical step. The colonization of cells from the periphery into the deeper regions of the scaffold is complex due to its microstructure. While these microstructures help preserve many native characteristics, they also limit the migration of chondrocytes, hindering their penetration into the scaffold [79, 80]. Moreover, the capacity for vascular preservation is an intriguing aspect of decellularization techniques. Although decellularization can maintain vascular ECM, this characteristic is less relevant in auricular engineering, as auricular cartilage is an avascular tissue [81]. Additionally, decellularized scaffolds may provoke adverse immune reactions due to the residual DNA from the original tissue. Therefore, evaluating their safety and immunogenicity in living organisms is crucial before clinical translation and application [82].

## Use of Additive Manufacturing for Personalized Scaffolds

Additive manufacturing methods are another proper technique for generating scaffolds (Fig. 6B and C). The manufacturing process begins with image acquisition. Computed tomography (CT) employs radiation to capture images in various cross-sections, which can then be processed into three-dimensional models. CT is widely available in hospitals, making this technology an accessible source for image acquisition. The images obtained require post-processing to generate a functional 3D model, necessitating a skilled operator to optimize the final morphology [25, 33]. An alternative method for image acquisition is the use of commercial 3D scanners. These scanning devices come equipped with specialized software that aligns data collected from different angles and distances relative to the object of interest. This capability enhances workflow, reduces operator-dependent variability, and makes the process more user-friendly [24, 25]. Unlike CT, commercial scanners are limited to external structures. This limitation is significant in ear regeneration, as it must account for non-cartilaginous structures such as the lobule, which can result in a thicker 3D model due to skin width. Additionally, the high costs associated with 3D scanning technology can be a barrier. An intriguing alternative is photogrammetric modeling, which allows for image acquisition using any camera, including those found in smartphones, and processing through specialized software. This approach reduces the need for expensive scanner purchases, and the widespread availability of smartphone technology makes it an appealing technique to explore [83]. Regardless of the acquisition technology employed, 3D models can be highly personalized. A process known as mirroring can be used to generate a contralateral ear for microtia patients, thereby enhancing facial symmetry [42, 45]. Once the 3D model is prepared, the next step is to print the ear according to CAD-CAM instructions, where the material is deposited layer by layer until the final scaffold is completed. [84]. Bioprinters or fused deposition modeling (FDM) printers are typically employed. The bioprinting process involves creating bio-inks to encapsulate cells within hydrogels to use in 3D structures. The concept of a method that allows for controlling cell types, ECM composition, and the spatial relationships of all components positions bioprinting as a powerful and up-and-coming tool for regenerating complex histological structures. Although bioprinting scaffolds offer many advantages, they lack structural stability. A significant limitation of this technique is its inability to provide adequate support for large or complex anatomical structures. Consequently, studies attempting to regenerate the auricle using this method have struggled to maintain the auricular shape, resulting in structural contraction and a loss of auricular morphology [85, 86]. To address this issue, combining bioprinting with polycaprolactone (PCL) scaffold synthesis has emerged as a promising solution [87]. Despite the successful implementation of this technique, the aesthetic outcomes have not met expectations, necessitating improvements in model accuracy to evaluate its potential for regenerating the human auricle [84]. An alternative to bio-inks is the use of biocompatible polymers. Various polymers can be utilized for FDM, with PCL being one of the most frequently employed due to its approval by the Food and Drug Administration (FDA) for clinical use [86]. In auricular reconstruction, a translational study was conducted using PCL in combination with polylactic acid (PLA) and polyglycolic acid (PGA). Initially, a computed tomography scan generated a 3D model of a healthy ear. Subsequently, a PCL mesh was printed, surrounded by unwoven PGA fibers, and coated with PLA. Finally, the scaffold was seeded with autologous chondrocytes obtained from the remnants of microtia cartilage. While the results represent a significant advancement, the retention of complex auricular morphology remains an unresolved challenge [42]. Therefore, future studies will be essential for manufacturing accurate, functional scaffolds, and exploring alternative materials will be crucial for developing improved scaffolds for auricular engineering.

## Perspectives and Conclusion

Current evidence identifies two principal sources of chondrocytes relevant to microtia reconstruction: residual microtia cartilage and healthy auricular cartilage. While microtia remnants offer an accessible and autologous cell source that aligns with the early stages of Nagata’s technique, their biological reliability remains questionable. The underlying etiology of microtia is still poorly understood, raising concerns that chondrocytes derived from these remnants may carry genetic, structural, or epigenetic abnormalities that impair their regenerative potential. In contrast, chondrocytes isolated from healthy auricular cartilage demonstrate more robust migratory and proliferative behavior, suggesting a higher functional suitability for tissue engineering. However, obtaining these cells poses ethical and technical challenges, particularly regarding donor site morbidity. This underscores the need to develop minimally invasive harvesting strategies that enable the collection and expansion of functionally competent chondrocytes without compromising healthy tissue.

Simultaneously, scaffold design is undergoing significant refinement. The field is moving beyond single-material scaffolds to hybrid fabrication strategies combining diverse biomaterials and manufacturing methods. This integrated approach aims to overcome the mechanical and biological limitations inherent to individual materials, improving scaffold performance in terms of biocompatibility, structural integrity, and support for cellular function. Nevertheless, a major unresolved limitation is the long-term maintenance of scaffold morphology and architecture *in vivo,* an essential factor for clinical translation. In conclusion, optimizing both chondrocyte sourcing and scaffold engineering is critical for advancing reliable, scalable, and functionally relevant tissue-engineered auricular constructs for treating microtia.

## Data Availability

All relevant data are within the paper.
